# Transaldolase inhibits CD36 expression by modulating glutathione-p38 signaling, exerting protective effects against macrophage foam cell formation: Transaldolase inhibits CD36 expression via GSH-p38 signaling

**DOI:** 10.3724/abbs.2023146

**Published:** 2023-08-01

**Authors:** Chengyi Li, Zihao Song, Pengyue Gao, Wei Duan, Xiu Liu, Sijia Liang, Quan Gong, Jiawei Guo

**Affiliations:** 1 Department of Immunology School of Medicine Yangtze University Jingzhou 434023 China; 2 Department of Oncology Jingzhou Hospital Affiliated to Yangtze University Jingzhou 434023 China; 3 Department of Cardiovascular Surgery Nanfang Hospital Southern Medical University Guangzhou 510515 China; 4 Department of Pharmacology and Cardiac & Cerebral Vascular Research Center Zhongshan School of Medicine Sun Yat-Sen University Guangzhou 510080 China

**Keywords:** CD36, foam cell formation, GSH, p38 MAPK, transaldolase

## Abstract

In atherosclerosis, macrophage-derived foam cell formation is considered to be a hallmark of the pathological process; this occurs via the uptake of modified lipoproteins. In the present study, we aim to determine the role of transaldolase in foam cell formation and atherogenesis and reveal the mechanisms underlying its role. Bone marrow-derived macrophages (BMDMs) isolated from mice successfully form foam cells after treatment with oxidized low-density lipoprotein (80 μg/mL). Elevated transaldolase levels in the foam cell model are assessed by quantitative polymerase chain reaction and western blot analysis. Transaldolase overexpression and knockdown in BMDMs are achieved via plasmid transfection and small interfering RNA technology, respectively. We find that transaldolase overexpression effectively attenuates, whereas transaldolase knockdown accelerates, macrophage-derived foam cell formation through the inhibition or activation of cholesterol uptake mediated by the scavenger receptor cluster of differentiation 36 (CD36) in a p38 mitogen-activated protein kinase (MAPK) signaling-dependent manner. Transaldolase-mediated glutathione (GSH) homeostasis is identified as the upstream regulator of p38 MAPK-mediated CD36-dependent cholesterol uptake in BMDMs. Transaldolase upregulates GSH production, thereby suppressing p38 activity and reducing the CD36 level, ultimately preventing foam cell formation and atherosclerosis. Thus, our findings indicate that the transaldolase-GSH-p38-CD36 axis may represent a promising therapeutic target for atherosclerosis.

## Introduction

Atherosclerotic cardiovascular disease is the major cause of mortality and morbidity worldwide. Excessive cholesterol deposition within the arterial intima is a significant contributor to the formation and development of atherosclerotic lesions, representing a chronic progressive disease [ 1, 2]. During atherogenesis, circulating monocytes transmigrate into the subendothelial connective tissue. Furthermore, they differentiate into macrophages, in which large amounts of cholesterol lipids accumulate through the uptake of oxidized low-density lipoprotein (oxLDL), a process mediated by scavenger receptors, such as scavenger receptor A (SR-A), cluster of differentiation 36 (CD36), and lectin-like oxLDL receptor-1 (LOX-1) [ 1, 3]. In contrast, adenosine triphosphate-binding cassette transporter A1 (ABCA1) and adenosine triphosphate-binding cassette transporter G1 (ABCG1) are the major cholesterol efflux transporters responsible for the efflux of cholesterol from macrophages to high‐density lipoproteins [4]. The imbalance in cholesterol influx and efflux leads to the accumulation of esterified cholesterol within cytosolic lipid droplets and the transformation of macrophages into foam cells [ 5 , 6]. Although the accumulation of lipid-laden (foam) cells in the arterial wall has been recognized as a critical step in the pathogenesis of atherosclerosis, the molecular mechanisms underlying this process are still not fully understood.

Transaldolase, a key enzyme of the non-oxidative pentose phosphate pathway (PPP), is encoded by *TALDO1*. Transaldolase indirectly controls metabolic flux through the PPP to generate nicotinamide adenine dinucleotide phosphate (NADPH) and indirectly regulates the intracellular glutathione (GSH) level in a cell type-specific manner [ 7– 10]. Notably, NADPH, a reducing factor generated from the PPP pathway, enzymatically reduces glutathione disulfide (GSSH) to GSH [11]. Furthermore, GSH depletion plays an important role in the development of atherosclerosis [ 12, 13]. P38 mitogen-activated protein kinases (p38 MAPK) can be selectively activated under low level of oxidative stress, which is potentiated by GSH depletion [14]; the protective effect mediated by GSH may be partly achieved through suppressing p38 MAPK signaling [15]. Furthermore, peroxisome proliferators–activated receptor γ (PPARγ), a member of a nuclear receptor superfamily, heterodimerizes with the retinoid X receptor (RXR) and activates the transcription of target genes through binding to the PPAR response elements (PPRE) [16]. Moreover, owing to the presence of a functional PPRE in *CD36* promoter, oxLDL can promote CD36 expression via PPARγ activation [17]. The interaction between p38 MAPK and PPARγ has been previously demonstrated; p38 MAPK activates and stabilizes PPARγ coactivator-1, and inhibition of p38 reduces PPARγ activity [18]. Yang *et al*. [13] reported that treatment with the GSH biosynthesis inhibitor buthionine sulfoximine (BSO) and N-acetyl cysteine (NAC), a precursor of GSH synthesis, accentuates and inhibits the oxLDL uptake of macrophages, respectively, via a CD36-dependent signaling pathway. In addition, elevated transaldolase expression is associated with pro-atherogenic phenotypes observed in oscillatory shear stress (OSS) intervention [19]. Together, the findings from these aforementioned studies suggest the potential association between transaldolase and the pathological process of atherosclerosis. However, thus far, no studies have been carried out to investigate the mechanism underlying the involvement of transaldolase in macrophage lipid metabolism.

In the present study, we explored the functional role of transaldolase in foam cell formation and the mechanism underlying this role by subjecting mouse-derived bone marrow-derived macrophages (BMDMs) to transaldolase overexpression or knockdown and then assessing the effects of altered transaldolase expression on foam cell formation. We also aimed to assess the mechanisms underlying these effects by analyzing cholesterol efflux, GSH contents, and oxLDL uptake and via western blot analysis and quantitative real-time polymerase chain reaction.

## Materials and Methods

### Materials and chemicals

The p38 inhibitor SB203580, NAC, and buthionine sulfoximine (BSO) were obtained from Sigma-Aldrich (St Louis, USA).

### Isolation of BMDMs from mice

Primary BMDMs were isolated from mice as described in a previous study [1]. All protocols involving animals were approved by the Ethics Committee of the Health Science Center of the School of Medicine at Yangtze University (Approval No. 202301002) and were performed in accordance with the Guide for the Care and Use of Laboratory Animals. Mice (C57BL/6 background) were obtained from Cyagen (Suzhou, China). All mice were housed under specific pathogen-free conditions in a temperature-controlled room under a 12-h light/dark cycle with *ad libitum* access to food and water. Only 8- to 10-week-old male mice (24–27 g) were included in the study. The mice were euthanized using sodium pentobarbital and their tibiae and femurs were collected. The marrows of these bones were flushed with phosphate-buffered saline (PBS) and filtered through a 70-μm strainer. The cells were seeded in Dulbecco’s Modified Eagle Medium (DMEM; Gibco, Waltham, USA) supplemented with 10% fetal bovine serum (FBS; Gibco), 1% HEPES, 1% penicillin/streptomycin, and 30% L929 conditioned medium (a supplier of M-CSF) for seven days to enable the differentiation into BMDMs.

### Cell lines and cell culture

The THP-1 cells were purchased from the Type Culture Collection of the Chinese Academy of Sciences (Shanghai, China) and cultured in RPMI-1640 medium (Gibco) containing 10% FBS and 1% penicillin-streptomycin in a humidified incubator maintained at 37°C under 5% CO _2_ conditions.

### Isolation and purification of LDL

Plasma (400 mL) collected from healthy donors was loaded into a dialysis bag through the semi-permeable membrane and concentrated into a final volume of 150 mL on ice with PEG20000. The plasma density was adjusted to 1.2 g/mL with KBr and was added into an ultracentrifugation tube mixed with a high-density liquid (density 1.063 g/mL) and a low-density liquid (1.020 g/mL). After centrifugation at 105000 *g* at 4°C for 5 h, a yellow substance, identified as LDL, located at an intermediate position between the high-density liquid and low-density liquid, was obtained. LDL was then placed in a dialysis bag and transferred to a dialysate comprising 20 mM Tris, pH 7.4, 50 mM NaCl, 300 μM EDTA, and 15 mM NaN _3_ for 24 h at 4°C. The dialysate was changed every 8 h, and the LDL was then sterilized by filtration through a 0.22-μm microporous membrane and stored at 4°C in the dark until use.

### Preparation of oxLDL

LDL (1 mL) was mixed with 50 μL of 100 μM CuSO _4_ (final concentration of 5 μM) and then incubated with sodium azide at 37°C for 24 h. Subsequently, the samples were dialyzed against phosphate buffer (pH 7.4) containing 200 μM EDTA for 24 h at 4°C to remove CuSO _4_. During this period, the solution was changed several times to remove residual EDTA. The oxLDL was sterilized by filtration through 0.22-μm membrane filters and protein concentration was then determined by BCA assay. Purified samples were stored at 4°C in the dark for future use.

### siRNA transfection

The sequences of siRNAs against mouse TALDO1, CD36, and the scrambled sequence were designed by OBiO Technology Corp (Shanghai, China). The target siRNA sequences were as follows: TALDO1 siRNA, 5′-AGGACAGAAUUCUCAUCAAGU-3′; CD36 siRNA, 5′-GGUUGUUCUACUUCCUUUAGC-3′; and scrambled (scr) siRNA, 5′-TTCTCCGAACGTGTCACGT-3′. First, the BMDMs were seeded into 6-well plates and transfected with the indicated siRNAs using the Lipofectamine™ 2000 transfection reagent (Invitrogen, Carlsbad, USA) in a serum‐free‐medium for 6 h according to the manufacturer’s protocols. Next, the transfection mixture was replaced by fresh complete DMEM containing 10% fetal calf serum (Gibco). After 48 h, western blot analysis was performed to examine the silencing efficiency of the siRNAs.

### Plasmid construction and transfection

Mouse TALDO1 and CD36 cDNA were amplified from the mouse cDNA library and cloned into a pcDNA3.1/Flag vector (Invitrogen). All the primers used for plasmid construction are listed in Supplementary Table S1. The transfection of the plasmids into BMDMs was performed using Lipofectamine™ 2000 reagent (Invitrogen) according to the manufacturer’s instructions. In brief, the BMDMs were first seeded into 6-well plates. Next, the DNA sample diluted in Opti-MEM medium was added to the Lipofectamine mixture which was also diluted in Opti-MEM medium. After 10 min, the DNA–lipid complex was added to the BMDMs in a dropwise manner, followed by incubation for 6 h. After this period, the medium was replaced by a complete medium, followed by incubation for 48 h. Western blot analysis was used to examine the overexpression efficiency of this plasmid.

### RNA isolation and quantitative real-time polymerase chain reaction (qPCR)

RNA isolation and quantitative real-time PCR (qPCR) were performed as described in previous studies [ 20, 21 ]. Briefly, the RNA samples were collected from BMDMs using TriZol (Invitrogen) according to the manufacturer’s instructions. Approximately 2 μg of total RNA was reverse transcribed into cDNA using a Reverse Transcription kit (Bio-Rad Laboratories, Hercules, USA). Individual real-time PCR was performed using the SYBR Green PCR Master Mix (Bio-Rad Laboratories) on a MyiQ Single Colour Real-time PCR Detection System (Bio-Rad Laboratories). The primer sequences used forqPCR are listed in Supplementary Table S2. The relative messenger RNA (mRNA) levels were calculated using the 2 ^-ΔΔCT^ method and *β-actin* was used as an internal reference.

### Protein extraction and western blot analysis

Western blot analysis was performed as described in previous studies [ 20, 21]. First, the BMDMs were washed with ice-cold PBS and resuspended in ice-cold radioimmunoprecipitation assay buffer containing a protease inhibitor cocktail (Roche, Basel, Switzerland) and phosphatase inhibitor (Roche) for 30 min. After centrifugation, the concentrations of proteins collected from the supernatants were measured using the Pierce BCA Protein Assay Kit (Thermo Fisher Scientific, Waltham, USA). After centrifugation for 10 min at 12,000 *g*, the supernatants were boiled at 95°C for 10 min in a sodium dodecyl sulfate (SDS) loading buffer. Aliquots of the protein samples were subjected to SDS-polyacrylamide gel electrophoresis; the resultant bands were transferred onto polyvinylidene fluoride membranes (Millipore, Billerica, USA). After being blocked with 5% nonfat milk at room temperature for 1 h, the membranes were probed with various primary antibodies overnight at 4°C and then incubated with the appropriate horseradish peroxidase-conjugated secondary antibodies for 1 h at room temperature. The protein bands were detected using an enhanced chemiluminescence reagent (Thermo Fisher Scientific). The images of the blots were captured using the ChemiDoc MP Imaging System (Bio-Rad). The β-actin protein served as the internal control. The antibodies used for the western blot analysis are listed in Supplementary Table S3.

### Oil red O staining

The BMDMs were transfected with siRNAs or plasmids and then incubated with 80 μg/mL oxLDL for 48 h to initiate the foam cell formation. Then, the cells were fixed with 4% paraformaldehyde at room temperature and stained with 0.3% oil red O (Sigma-Aldrich) dissolved in 60% isopropanol for 10 min at room temperature. Lipid droplets were visualized using a light microscope (Olympus, Tokyo, Japan). Additionally, the quantitative analysis of oil red O staining intensity was performed using the ImageJ software.

### Analysis of l,l′-dioctadecyl-3,3,3′,3′-tetramethyl-indocarbocyanine perchlorate (Dil)-oxLDL uptake

The oxLDL uptake by BMDMs was analyzed by labeling oxLDL with Dil as described in a previous study [1]. The BMDMs were incubated with 10 μg/mL Dil-oxLDL for 4 h at 37°C, and fixed with 4% paraformaldehyde solution for 10 min, followed by staining with 4′,6-diamidino-2-phenylindole to visualize the nuclei. After several times wash with PBS, the cells were photographed under a Zeiss LSM800 confocal microscope (Zeiss, Oberkochen, Germany). Finally, the fluorescence intensity of the samples was quantified using the ImageJ software.

### Cholesterol efflux analysis

Cholesterol efflux was measured using the cholesterol efflux assay kit (Abcam, Cambridge, UK) following the manufacturer’s instructions. Briefly, BMDMs were incubated with fluorescently labeled cholesterol overnight. High-density lipoprotein was used as an acceptor of cholesterol in this assay. Cholesterol efflux was estimated as a percentage of fluorescence in the supernatant compared to the sum of the fluorescence of the supernatant and cell lysate.

### Analysis of GSH contents

According to the manufacturer’s protocol, the total GSH level in the BMDMs was measured using a Glutathione Assay Kit (Cayman Chemical, Ann Arbor, USA). Briefly, an equal number of BMDMs were seeded in a 10-cm dish. The cells were washed twice with PBS and detached using a rubber scraper. Then, they were suspended in 1 mL of ice-cold PBS. The cells were pelleted by centrifugation at 400 *g* for 5 min at 4°C and resuspended in 100 μL of 2-(N-morpholino) ethanesulfonic acid (MES) buffer (50 mM MES, 1 mM EDTA, pH 6). The samples were immediately frozen in liquid nitrogen and thawed on ice for two repeated cycles. The supernatants were collected after centrifugation at 12,000 *g* for 15 min at 4°C and then deproteinated with 5% metaphosphoric acid (Sigma-Aldrich), followed by vortexing and centrifugation at 3000 *g* for 2 min. Finally, triethanolamine was added to the collected supernatants. The total GSH contents in the resulting supernatants were measured after treatment with the kit reagents in a 96-well plate. After incubation for 25 min in the dark, the absorbance of the samples was measured at 405 nm using a microplate reader (BioTek, Winooski, USA).

### Statistical analysis

Data were expressed as the mean±standard deviation of the indicated number of independent experiments. A two-tailed *t*-test was used to compare the difference between two groups. The comparisons among three or more groups were assessed by one-way analysis of variance (ANOVA), followed by Tukey’s post hoc test. Two-way ANOVA was considered for analyzing two independent variables, followed by Tukey’s post hoc test for multiple comparisons. All statistical analyses were performed using the Statistical Package for the Social Sciences (SPSS) statistical software (version 22.0; SPSS, Armonk, USA) and GraphPad Prism software (version 9.0; GraphPad Prism software, La Jolla, USA). *P*<0.05 was considered statistically significant.

## Results

### Transaldolase is upregulated in macrophages subjected to atherogenic stimuli

**Figure 1:**
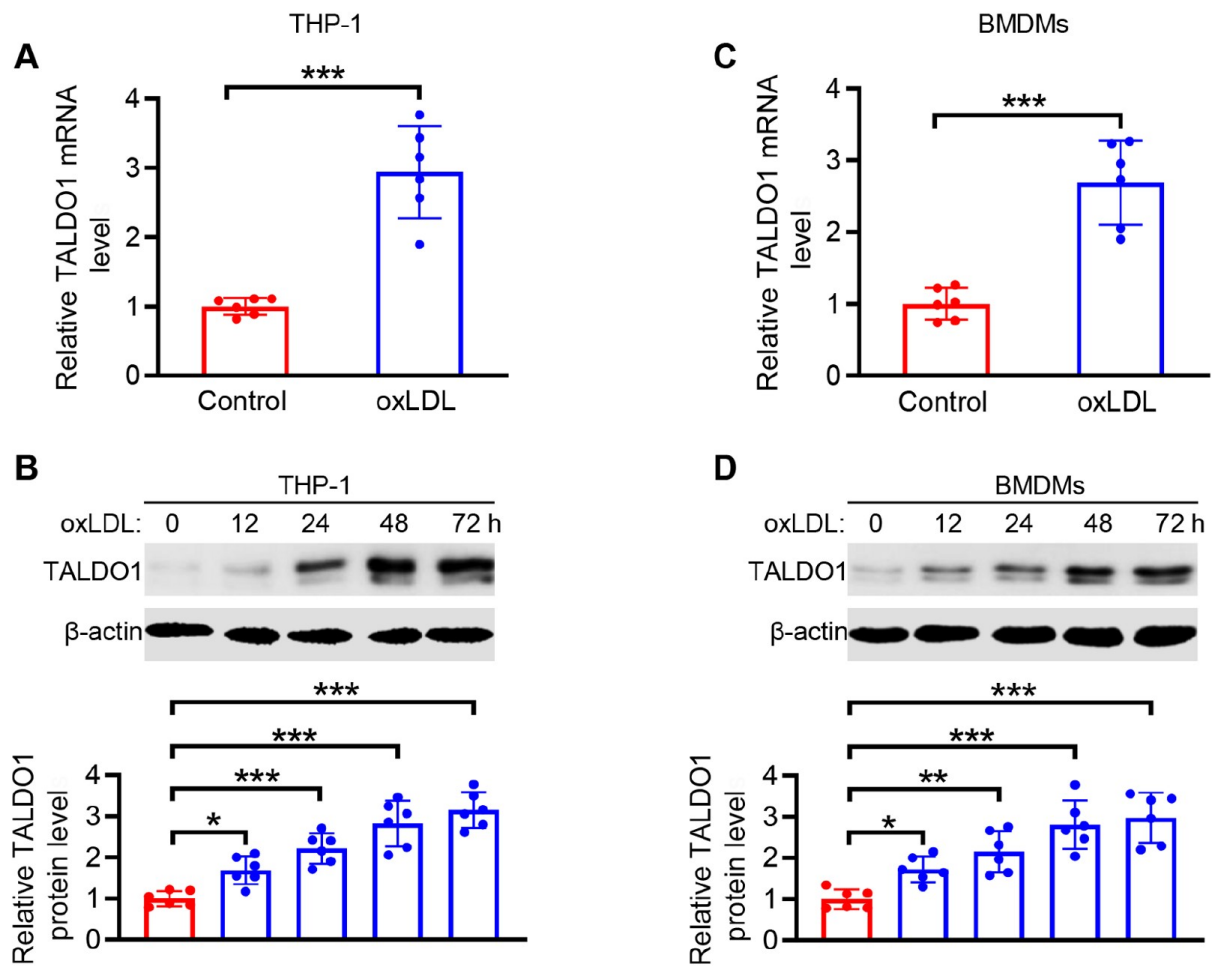
Figure 1 Transaldolase expression is upregulated in macrophages subjected to atherogenic stimuli (A,B) qPCR analysis of TALDO1 mRNA expression (A) and western blot analysis of transaldolase protein expression (B) in THP-1 macrophages treated with oxLDL (80 μg/mL) for 48 h. (C,D) Levels of TALDO1 mRNA (C) and protein (D) in bone-marrow derived macrophages (BMDMs) treated with oxLDL (80 μg/mL) for the indicated time periods. n=6, *P<0.05, ** P<0.01, ***P<0.001.

A crucial step in the development of atherosclerosis is the formation of macrophage-derived foam cells. Tissue macrophages will transform into foam cells upon exposure to oxLDL [1]. To determine the potential role of transaldolase in foam cell formation in macrophages, we first measured the expression of transaldolase in both oxLDL-treated human THP-1 macrophages and primary BMDMs isolated from C57BL/6J mice. The mRNA and protein levels of TALDO1 gradually increased with the increase in the duration of treatment with oxLDL ( Figure 1A–D). Concurring with these results, enhanced TALDO1 expression was also confirmed in mouse BMDMs isolated from Apoe ^–/–^ mice fed with a high-fat diet (HFD) ( Supplementary Figure S1), suggesting a potential role of transaldolase in foam cell formation and atherosclerosis development.

### Transaldolase suppresses oxLDL-induced formation of foam cells derived from macrophages

**Figure 2:**
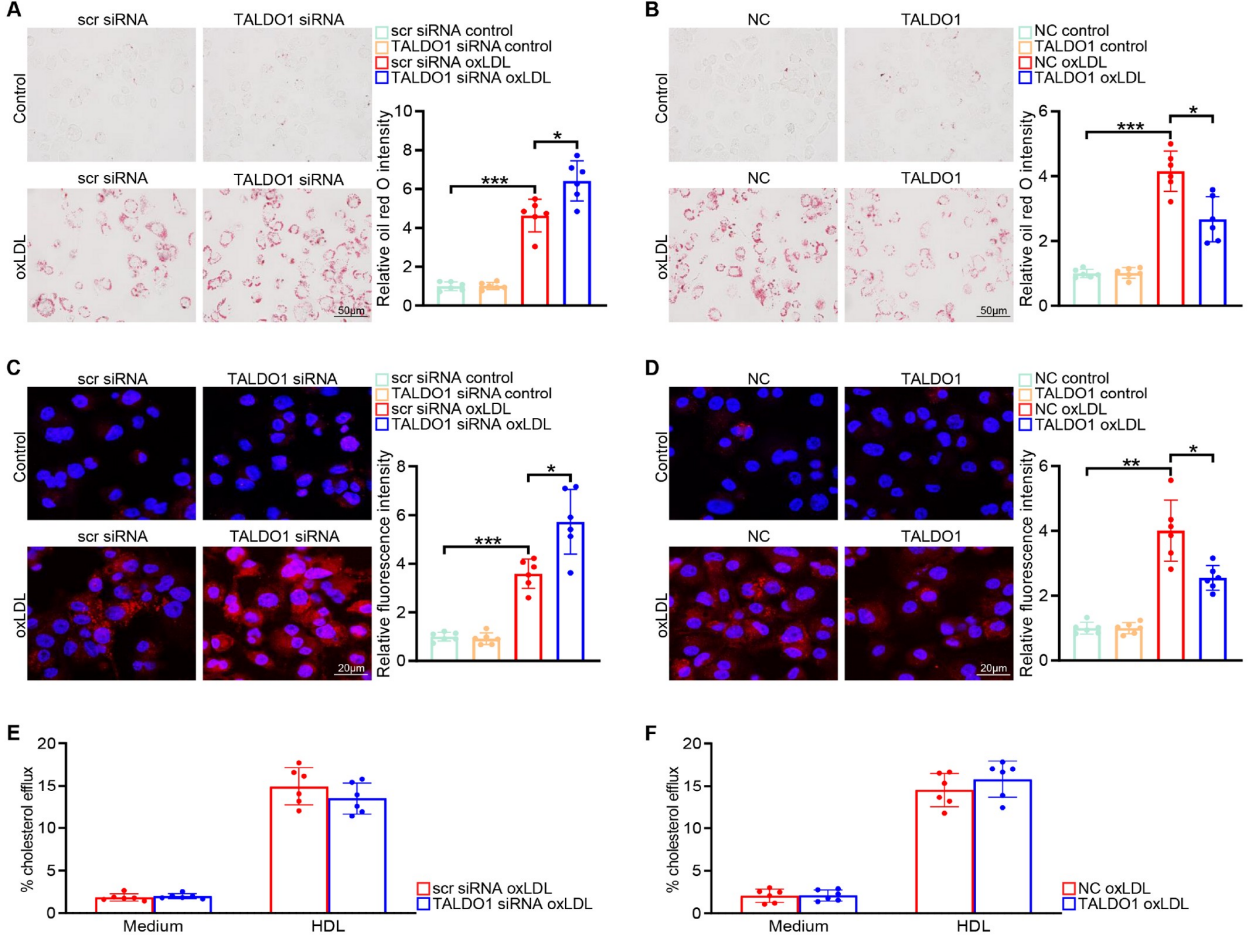
Figure 2 Transaldolase suppresses oxLDL-induced formation of macrophage-derived foam cells (A) Representative images of oil red O staining of BMDMs transfected with scrambled (scr) siRNA or TALDO1 siRNA in the presence/absence of oxLDL (80 μg/mL) stimulation for 48 h (left panel) and quantification of oil red O staining intensity (right panel). (B) Representative images of oil red O staining of BMDMs transfected with negative control (NC) vector or TALDO1-overexpression plasmid in the presence/absence of oxLDL (80 μg/mL) stimulation for 48 h (left penal) and quantification of oil red O staining intensity (right panel). (C,D) BMDMs transfected with scr siRNA and TALDO1 siRNA (C) or with NC vector and TALDO1-overexpression plasmid (D) were treated without or with oxLDL (80 μg/mL) for 48 h, followed by incubation with Dil-oxLDL (10 μg/mL) for 4 h. Dil-oxLDL uptake was examined by confocal microscopy (left panel) and Dil-oxLDL-derived red fluorescence intensity was quantified (right panel). (E,F) Representative experiment analyzing cholesterol efflux to high-density lipoprotein from BMDMs transfected with scr siRNA and TALDO1-targeting siRNA (E) or NC vector and TALDO1-overexpression plasmid (F) after oxLDL (80 μg/mL) stimulation for 48 h. n=6, *P<0.05, **P<0.01, ***P <0.001.

The increased expression of transaldolase during the transformation of macrophages into foam cells prompted us to investigate the regulatory role of transaldolase in this process. Therefore, the *TALDO1* gene was knocked down or overexpressed by transfection with TALDO1 siRNA or TALDO1-overexpression plasmid, respectively. The efficiency of *TALDO1* knockdown or overexpression was confirmed by western blot analysis ( Supplementary Figure S2A,B). Oil Red O staining revealed that lipid accumulation in BMDM-derived foam cells was higher in the TALDO1 siRNA group than in the scr siRNA group, but was lower in the TALDO1 overexpression group than in the NC group ( Figure 2A,B). Importantly, foam cell development depends on imbalance of intracellular cholesterol influx (uptake of oxLDL) and the efflux of cholesterol from foam cells [22]. Therefore, we further investigated whether the suppressed uptake of oxLDL or amplified efflux of cholesterol or both account for the suppression of the formation of foam cells derived from TALDO1-overexpressing BMDMs. We performed a Dil-labeled oxLDL uptake assay and examined the lipid uptake by confocal microscopy. *TALDO1*-silenced BMDMs displayed a higher fluorescent intensity than the BMDMs transfected with scr siRNA, suggesting that *TALDO1* knockdown promoted Dil-oxLDL uptake by BMDMs ( Figure 2C). In contrast, the TALDO1-overexpressing BMDMs exhibited the opposite results ( Figure 2D). Subsequently, using a cholesterol efflux assay kit, we assessed whether transaldolase expression affects cholesterol efflux; however, we did not observe notable differences between *TALDO1*-silenced BMDMs and scr-siRNA-transfected cells or between TALDO1-overexpressing BMDMs and BMDMs transfected with the null overexpression vector (NC group) ( Figure 2E,F). Together, these results suggest that transaldolase suppresses foam cell formation by decreasing the oxLDL uptake.

### Transaldolase suppresses macrophage cholesterol uptake and foam cell formation via the inhibition of CD36 expression

**Figure 3:**
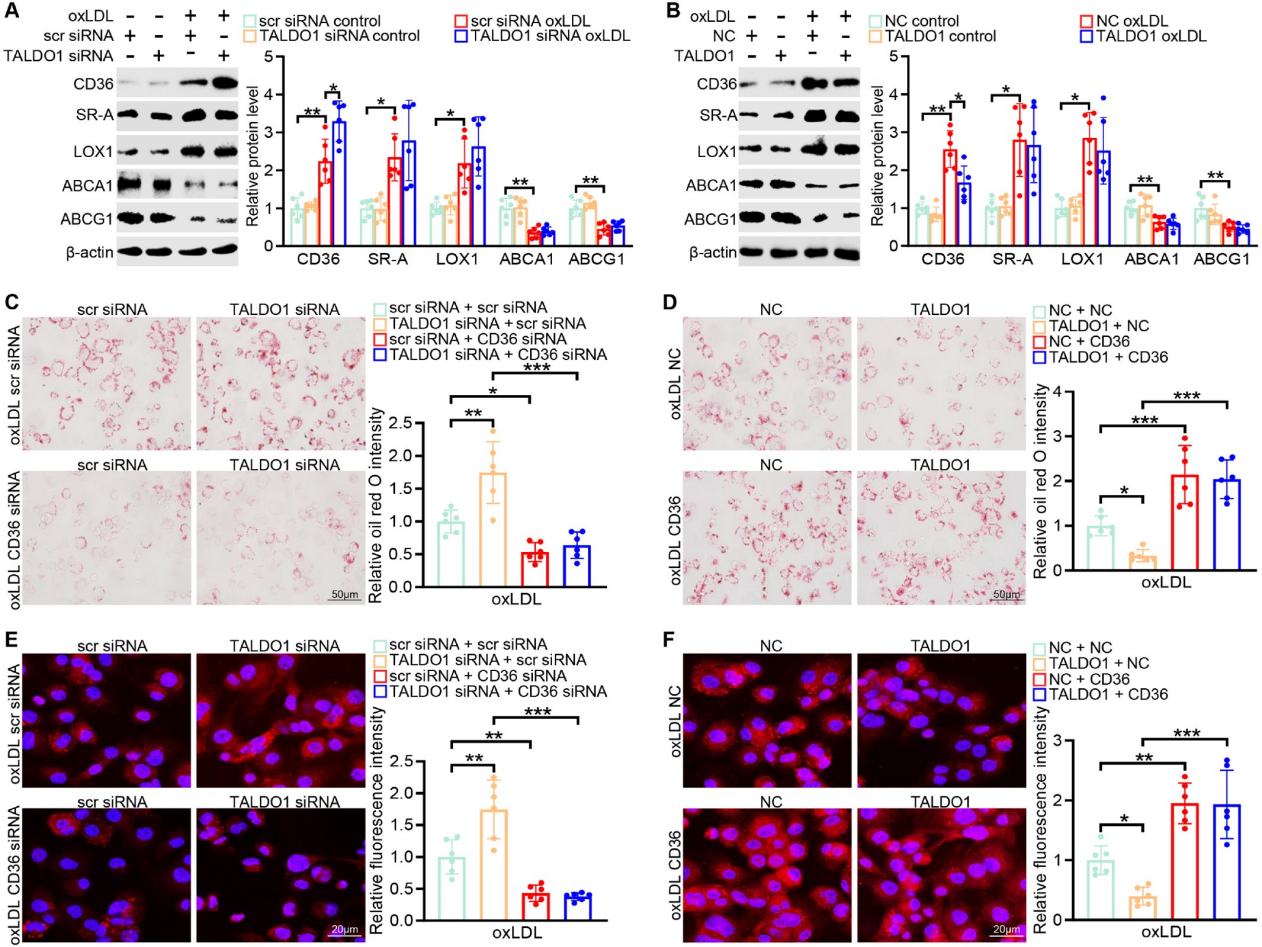
Figure 3 Transaldolase suppresses macrophage cholesterol uptake and foam cell formation via the inhibition of CD36 expression (A) CD36, SRA, LOX1, ABCA1, and ABCG1 protein levels in BMDMs transfected with scr siRNA or TALDO1 siRNA before oxLDL (80 μg/mL) treatment for 48 h. (B) CD36, SRA, LOX1, ABCA1, and ABCG1 protein levels in BMDMs treated with NC vector or TALDO1 plasmid and then subjected to oxLDL (80 μg/mL) treatment for 48 h. (C,E) Representative images of oil red O staining (C) and Dil-oxLDL uptake (E) in BMDMs pretreated with scr siRNA or CD36 siRNA, followed by incubation with scr siRNA or TALDO1 siRNA upon oxLDL (80 μg/mL) stimulation for 48 h. (D,F) Negative control vector- or TALDO1-overexpression plasmid-transfected BMDMs pre-incubated with an NC vector or CD36 plasmid were challenged with oxLDL (80 μg/mL) for 48 h and then subjected to oil red O staining (D) and Dil-oxLDL uptake assay (F). n=6, *P<0.05, **P<0.01, *** P<0.001.

We determined the mechanism underlying the transaldolase-mediated suppression of cholesterol uptake. Macrophage scavenger receptors, such as CD36, SR-A, and LOX-1, mediate the uptake of oxLDL [3]. Transfection with the TALDO1 siRNA or TALDO1-overexpression plasmid significantly facilitated or attenuated the expression of CD36, respectively, after oxLDL challenge in BMDMs. However, it exerted no marked effect on SR-A or LOX-1 expression ( Figure 3A,B). In addition, the transcription level of CD36 was further enhanced by *TALDO1* knockdown whereas it was hampered by TALDO1 overexpression ( Supplementary Figure S3). Consistent with the cholesterol efflux assay results, TALDO1 did not alter the expressions of ABCA1 and ABCG1, two efflux transporters responsible for cholesterol efflux [4] ( Figure 3A,B). An increased expression of CD36 was observed in the BMDMs of Apoe ^–/–^ mice fed with HFD ( Supplementary Figure S1), which was consistent with the results of previous studies [1]. To further ascertain the functional importance of CD36 in oxLDL uptake in response to transaldolase, TALDO1 siRNA- or TALDO1-overexpression plasmid-transfected BMDMs were pre-incubated with CD36 siRNA or CD36-overexpression plasmid, respectively. Simultaneously, the efficiency of *CD36* knockdown or overexpression was confirmed by western blot analysis ( Supplementary Figure S4). As expected, silencing of *TALDO1* in BMDMs remarkably augmented foam cell formation, as indicated by the results of the oil red O staining and oxLDL uptake analyses, which demonstrated increased levels of lipid droplets and oxLDL uptake, respectively; these effects were specifically abrogated by pretreatment with the CD36 siRNA ( Figure 3C,E). Moreover, treatment with the CD36-overexpression plasmid increased foam cell formation and oxLDL uptake, showing effects comparable to those seen in case of BMDMs transfected with the negative control or the TALDO1-overexpression plasmid ( Figure 3D,F). Taken together, these data indicate that transaldolase prevents oxLDL uptake in BMDMs and foam cell formation through the downregulation of CD36 expression.

### Transaldolase suppresses CD36-mediated cholesterol uptake via the p38 mitogen-activated protein kinase (MAPK) pathway

**Figure 4:**
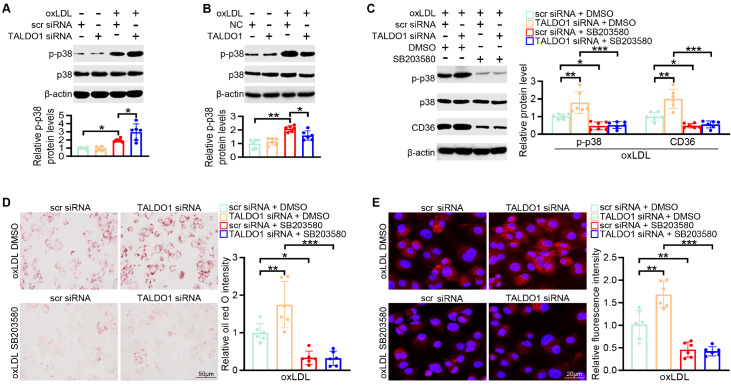
Figure 4 Transaldolase mitigates CD36-mediated cholesterol uptake via the p38 MAPK pathway (A,B) Western blot analysis of p-p38 expression in BMDMs treated with scr siRNA and TALDO1 siRNA (A) or an NC vector or TALDO1-overexpression plasmid (B) after treatment with oxLDL (80 μg/mL) for 48 h. (C) BMDMs were pre-incubated with the p38 inhibitor SB203580 (10 μM) for 3 h and then transfected with scr siRNA and TALDO1 siRNA before oxLDL (80 μg/mL) incubation for 48 h. The CD36 protein expression was then determined. (D,E) BMDMs were pretreated with or without SB203580 (10 μM) for 3 h, followed by transfection with scr siRNA and TALDO1 siRNA before oxLDL (80 μg/mL) treatment for 48 h. Oil-red O staining was performed to examine foam cell formation (D). Furthermore, fluorescence confocal microscopy was carried out to examine the uptake of Dil-oxLDL (E). n=6, * P<0.05, **P<0.01, ***P <0.001.

Three major members of the mitogen-activated protein kinase family, p38 MAP kinase (p38), extracellular signal-regulated kinase (ERK), and c-Jun N-terminal kinase (JNK), serve as vital elements in regulating CD36 expression [23]. The administration of oxLDL promoted the phosphorylation of p38, ERK, and JNK, and increased phosphorylation of p38 was also observed in Apoe ^–/–^ mice fed with HFD ( Supplementary Figure S1). However, only p38 activation was further enhanced by *TALDO1* knockdown; moreover, p38 activation was blocked by TALDO1 overexpression ( Figure 4A,B). However, ERK or JNK activation, as indicated by the levels of their phosphorylation, showed no difference in TALDO1 siRNA- or TALDO1-overexpression plasmid-transfected BMDMs ( Supplementary Figure S5A,B). To further explore whether p38 is involved in the transaldolase-mediated regulation of CD36 expression, we pretreated BMDMs with the p38 inhibitor SB203580. The inhibition of p38 markedly reversed the *TALDO1*-knockdown-dependent upregulation of CD36 mRNA and protein expression ( Figure 4C and Supplementary Figure S6). Moreover, treatment with SB203580 suppressed oxLDL-induced foam cell formation and cholesterol uptake, showing effects comparable to those seen in case of TALDO1 siRNA- and scr siRNA-treated BMDMs ( Figure 4D,E). These results indicate that p38-CD36 signaling is required for transaldolase-mediated foam cell formation.

### GSH is required for the transaldolase-mediated inhibition of p38 activity

**Figure 5:**
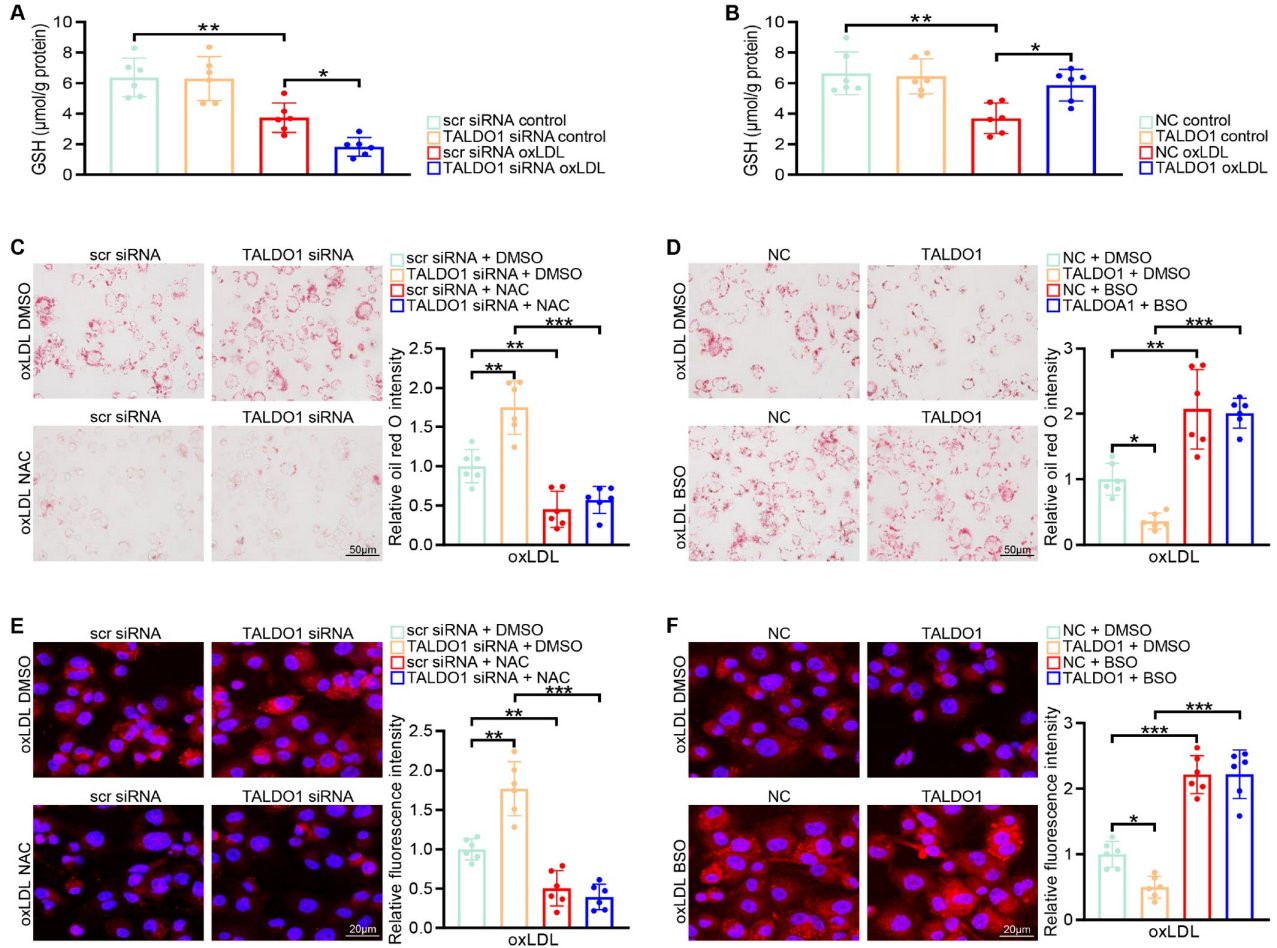
Figure 5 GSH is required for transaldolase-mediated inhibition of p38 activity (A,B) GSH levels were measured in BMDMs treated with scr siRNA and TALDO1 siRNA (A) or an NC vector and TALDO1-overexpression plasmid (B) after treatment with oxLDL (80 μg/mL) for 48 h. (C,E) Scr siRNA- and TALDO1 siRNA-transfected BMDMs were pretreated with or without NAC (10 mM) for 16 h. After treatment, the lipid content was assessed by oil red O staining (C) or Dil-oxLDL uptake analysis (E) after oxLDL administration for 48 h. (D,F) Representative images of oil red O staining (D) and Dil-oxLDL uptake (F) in BMDMs pretreated with or without buthionine sulfoximine (5 μM) for 16 h, followed by incubation with an NC vector or TALDO1-overexpression plasmid before oxLDL (80 μg/mL) stimulation for 48 h. n=6, *P<0.05, **P<0.01, *** P<0.001.

Previous studies have reported that NAC, a precursor of GSH, inhibits the activation of p38 [ 24, 25 ], suggesting that GSH acts on upstream signaling pathways for p38 inhibition. Additionally, transaldolase deficiency in mouse livers was reported to result in GSH depletion [9]. These phenomena support the existence of a bridge between transaldolase expression and the inhibition of p38 activity. In the present study, the GSH level in BMDMs treated with TALDO1 siRNA was lower than that in BMDMs treated with scr siRNA. In contrast, transaldolase overexpression increased GSH contents in BMDMs ( Figure 5A,B). To further examine the role of GSH in mediating the function of transaldolase in foam cell formation and cholesterol uptake, BMDMs transfected with TALDO1 siRNA or TALDO1-overexpression plasmid were pre-incubated with a GSH precursor (NAC) or GSH synthesis inhibitor (BSO), followed by oxLDL challenge. The facilitated effects of *TALDO1* knockdown on foam cell formation and oxLDL uptake in BMDMs were dramatically abolished by the pharmacological induction of GSH activity. However, the pharmacological inhibition of GSH resulted in increased foam cell formation and oxLDL uptake in BMDMs transfected with either TALDO1-overexpression plasmid or the negative control plasmid ( Figure 5C–F). Furthermore, the administration of NAC reversed the activation of p38 activity and upregulation of CD36 expression observed in TALDO1 siRNA-treated BMDMs after oxLDL challenge. However, treatment with the GSH inhibitor abolished the protective effects of transaldolase on foam cell formation, as demonstrated by the increase in p38 activity and CD36 expression ( Supplementary Figure S7A,B). These results indicate that GSH contributes to the protective effects of transaldolase against foam cell formation.

## Discussion

The accumulation of lipid-laden macrophage foam cells in the arterial wall is a key step in early atherogenesis. Modified LDLs, such as oxLDL, strongly induce the formation of macrophage foam cells and the progression of atherosclerotic lesion formation [ 1 , 26]. Therefore, in the present study, we attempted to explore the role of transaldolase in foam cell formation. Our data revealed that the level of transaldolase was highly elevated in macrophages after oxLDL administration. Knockdown of the transaldolase gene remarkably accentuated the atherogenic process of lipid oxLDL-induced formation of mouse primary BMDM-derived foam cells. However, these effects were markedly suppressed by transaldolase overexpression. Mechanistically, the beneficial effects of transaldolase overexpression on suppressing foam cell formation largely depend on the inhibition of p38 MAPK-CD36-mediated cholesterol uptake in a GSH-dependent manner.

Transaldolase expression is upregulated or downregulated depending on the cellular state under different physiological and pathological conditions [ 27, 28]. Notably, a previous study has revealed that transaldolase is highly expressed in response to OSS-induced atherosclerosis, as assessed by integrating metabolomic and proteomic analyses and via qPCR analyses [19]. Consistent with these previous findings, in this study, we observed that transaldolase expression was increased after oxLDL stimulation. These findings suggest that transaldolase expression may increase during the development of atherosclerosis caused by different genetic and clinical factors. However, whether the elevation of transaldolase expression is merely an epiphenomenon that occurs as a result of atherosclerosis or plays a role in this disease remains unclear. In the present study, we showed that transaldolase overexpression substantially inhibits the formation of macrophage-derived foam cells. Intriguingly, the protective function of transaldolase against oxLDL-dependent phenotypic modulation in macrophages and its dramatic upregulation in the initial critical stage of atherosclerosis appear to represent a paradox. This is a common phenomenon frequently reported to be associated with the upregulation of protective factors under conditions of stress stimulation [ 29 , 30]. Thus, the altered transaldolase expression in atherosclerosis cannot directly predict the macrophage phenotype but suggests the possible participation of transaldolase in atherosclerosis progression. However, there may be other upregulated proteins in the foam cell formation that play a negative feedback role in maintaining macrophage homeostasis.

Lipid accumulation in macrophages and foam cell formation are resulted from a disturbance in the homeostasis between lipid uptake and cholesterol efflux [31]. Therefore, in the present study, we first measured the influence of transaldolase on the expressions of three major scavenger receptors responsible for oxLDL uptake (CD36, LOX-1, and SR-A) [3]. Our results clearly demonstrate that transaldolase overexpression blocks CD36 expression without SR-A and LOX-1 upregulation during foam cell formation. Furthermore, we also investigated whether transaldolase affects cholesterol efflux and found that transaldolase does not modify the expressions of cholesterol efflux transporters (ABCA1 and ABCG1) or cholesterol efflux. In conclusion, our data show that CD36 may be the primary receptor responsible for foam cell formation induced by transaldolase downregulation.

The MAPK pathway mediates many diverse biological processes, including proliferation, differentiation, inflammation, and autophagy. It also plays a critical role in the pathogenesis of atherosclerosis [23]. Activation of p38 positively regulates the expression of CD36 and foam cell formation [ 23 , 32]. Although the phosphorylation of ERK, JNK, and p38 in BMDMs increases after stimulation with oxLDL, downregulation or overexpression transaldolase only affects the phosphorylation of p38; it does not affect the ERK or JNK signaling pathway. Importantly, our results indicate that the CD36 expression can also be suppressed by a specific p38 inhibitor, which is consistent with the experimental findings reported in other previous studies [ 23, 32]. Notably, in the present study, oxLDL uptake induced by transaldolase downregulation was abolished under exposure to the p38 inhibitor SB203580, indicating that the p38 MAPK signaling pathway may be responsible for the CD36 upregulation mediated by transaldolase downregulation.

Transaldolase functions are distinct between different tissues and cell types. Transaldolase activity profoundly affects the balance between the two branches of PPP, and the ultimate production of NADPH and GSH [33]. For instance, transaldolase overexpression increases GSH level in the liver tissues [9], but reduces GSH production in Jurkat lymphocytes and H9 human T cells [ 7, 8]. PPP, a major collateral pathway for glucose metabolism, has two unique functions: supplying ribose-5-phosphate (R5P) for the synthesis of nucleotides, DNA and RNA, and generating NADPH as a substrate for many biosynthetic reactions [33]. NADPH mediates the conversion of oxidized glutathione (GSSG) to reduced glutathione (GSH) [33]. The PPP consists of two branches: an irreversible oxidative branch, which primarily depends on glucose 6-phosphate dehydrogenase (G6PD), and a reversible non-oxidative branch. Transaldolase is the key rate-limiting enzyme of the non-oxidative branch. While the oxidative branch of the PPP is known to be recognized as the source of R5P and NADPH, eventually mediating regeneration of GSH, transaldolase-deficient mice or human lymphoblasts display NADPH depletion due to the accumulation of sedoheptulose 7-phosphate (S7P) and the failure to recycle ribose 5-phosphate (R5P) into G6P through the non-oxidative branch. Reduced NADPH production leads to secondary depletion of GSH [ 9, 34]. Our data suggest that GSH production indirectly mediated by transaldolase in BMDMs is comparable to that in liver tissues [9]. However, the effects of the suppression of transaldolase on promoting GSH production may be attributed to the diminution of G6PD and 6-phosphogluconate dehydrogenase (6PGD) activities in Jurkat and H9 human T cell lines [ 7, 8]. Thus, the effect of TALDO1 on GSH production may be cell type-specific, context-dependent, or both. The cell type-specific differences in the impact of transaldolase on enzyme activity involved in oxidative branch of the PPP and GSH output may be dependent on forward or reverse reactions catalyzed by transaldolase. Moreover, the depletion of GSH level by glutamine starvation or BSO treatment leads to the augmentation of p38 MAPK activity [35]. In contrast, increased GSH level induced by NAC inhibits this biological behavior [25], suggesting that GSH is a central hub in a molecular network for mediating transaldolase and p38 MAPK signaling in BMDMs. Consistent with this, NAC treatment suppresses, whereas BSO administration promotes, the transaldolase-mediated regulation of p38 activity. These findings indicate that GSH is crucial for transaldolase-mediated downstream signaling in macrophages. However, previous studies have shown that GSH is a major contributing factor in cellular antioxidant activity [13], and the GSH-dependent antioxidant system has been implicated in regulating foam cell formation and development of atherosclerosis [ 12, 36]. Thus, further research is needed to explore whether other factors or pathways are involved in GSH regulation of atherosclerosis development.

In conclusion, to the best of our knowledge, this is the first to provide evidence that transaldolase serves as a protective factor against foam cell formation and atherogenesis by downregulating CD36 expression and cholesterol uptake through the GSH-p38 MAPK signaling pathway. Our findings provide novel insights into the role of transaldolase in foam cell formation and identify it as a promising therapeutic target for preventing the progression of atherosclerotic vascular disease.

## Supplementary Data

Supplementary Data is available at *Acta Biochimica et Biophysica Sinica* online.

## Supporting information

085Supplementary_Material
