# Supplementary material for: Transaldolase inhibits CD36 expression by modulating glutathione-p38 signaling, exerting protective effects against macrophage foam cell formation: Transaldolase inhibits CD36 expression via GSH-p38 signaling
Source: Acta Biochim Biophys Sin (Shanghai). 2023 Aug 1;55(9):1496–505. doi: 10.3724/abbs.2023146 (PMC10520467; doi:10.3724/abbs.2023146)
Supplement: 085Supplementary_Material [file 085Supplementary_Material.pdf]

Supplementary Materials

Supplementary Table S1. Sequences of primers used for plasmid construction

Gene	Forward primer (5′→3′)	Reverse primer (5′→3′)
TALDO1	CGCAAATGGGCGGTAGGCGTG	CTTGCCCCTTGCTCCATACCAC
CD36	CGCAAATGGGCGGTAGGCGTG	TTATTAGGAAAGGACAGTGGG

Supplementary Table S2. Seuqences of primers used for qPCR analysis

Gene	Forward primer (5′→3′)	Reverse primer (5′→3′)
TALDO1 (human)	CTCACCCGTGAAGCGTCAG	GTTGGTGGTAGCATCCTGGG
TALDO1 (mouse)	CGATGAATACAAGCCCCAAGAT	AAGGAAAGCCTTGCATCAACT
β-actin (human)	GCTTCTCCTTAATGTCACGC	CCCACACTGTGCCCATCTAC
β-actin (mouse)	TATTGGCAACGAGCGGTTCC	GGCATAGAGGTCTTTACGGATGTC
CD36 (mouse)	ATGGGCTGTGATCGGAACTG	TTTGCCACGTCATCTGGGTTT

Supplementary Table S3. Antibodies used for western blot analysis

Antibodies	Source	Cat NO.	Dilution
β-actin	Proteintech	20536-1-AP	1:2000
TALDO1	Abcam	ab137629	1:1000
CD36	Proteintech	18836-1-AP	1:1000
SRA	Abcam	ab151707	1:1000
LOX1	Proteintech	11837-1-AP	1:1000
ABCA1	Abcam	ab18180	1:500
ABCG1	Abcam	ab52617	1:500
p-p38	Cell Signaling Technology	4511	1:1000
p38	Cell Signaling Technology	9212	1:1000
p-JNK	Santa Cruz Biotechnology	sc-12882	1:500
JNK	Santa Cruz Biotechnology	sc-7345	1:500
p-ERK	Cell Signaling Technology	9101	1:1000
ERK	Cell Signaling Technology	9102	1:1000

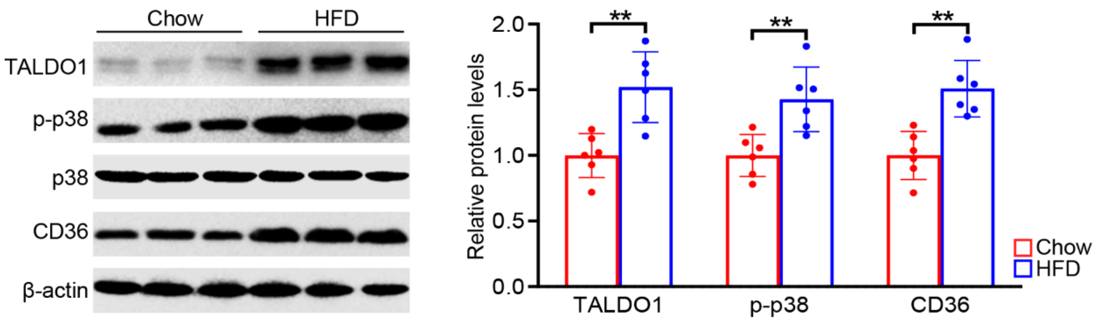

Supplementary Figure S1. Changes in protein expressions of TALDO1, p-p38, and CD36 in BMDMs isolated from Apoe$^{-/-}$ mice fed with HFD Representative western blots showing the TALDO1, p-p38, and CD36 levels in BMDMs derived from Apoe$^{-/-}$ mice fed with the chow diet or HFD for 12 weeks (*n*=6, ***P*<0.01).

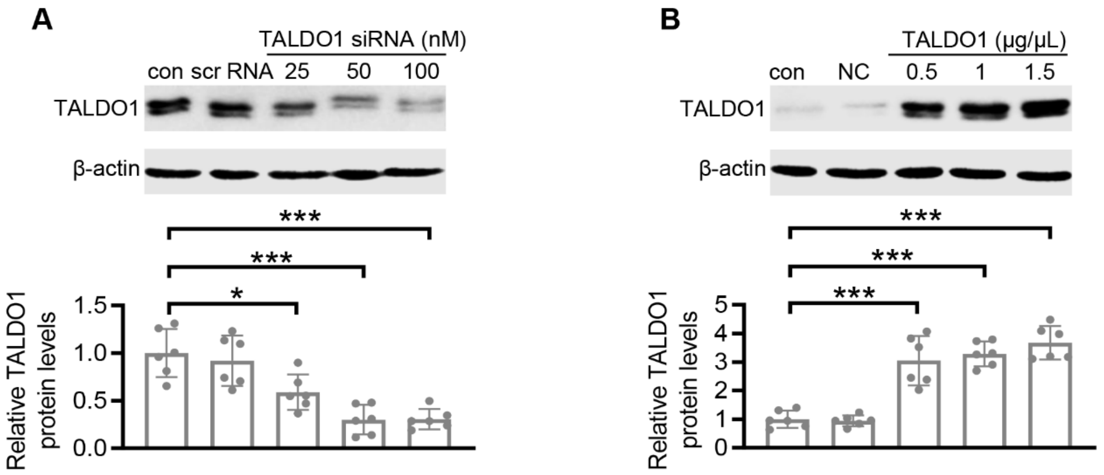

Supplementary Figure S2. Verification of *TALDO1* knockdown and overexpression in BMDMs (A) Western blot analysis was used to detect the efficiency of TALDO1 siRNA interference. (B) Transfection efficiency was verified after transfection with the TALDO1 overexpression plasmid. Therefore, a concentration of 50 nM siRNA and a concentration of 0.5 μg/μL plasmid were selected for subsequent experiments (*n*=6, **P*<0.05, ****P*<0.001).

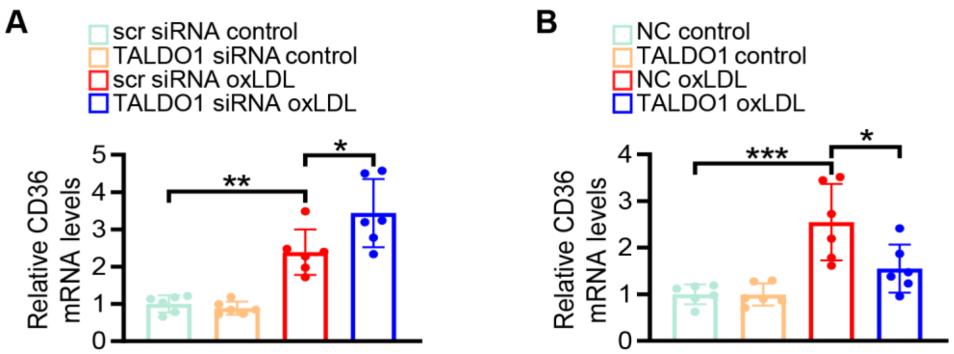

Supplementary Figure S3. Effects of transaldolase on mRNA levels of CD36 in BMDMs (A,B) CD36 mRNA levels in BMDMs incubated with scrambled (scr) siRNA and TALDO1 siRNA (A) or negative control vector (NC) and TALDO1 plasmid (B) after exposure to oxidized low-density lipoprotein (oxLDL) (80 μg/mL) for 48 h (n=6, *P<0.05, **P<0.01, ***P<0.001).

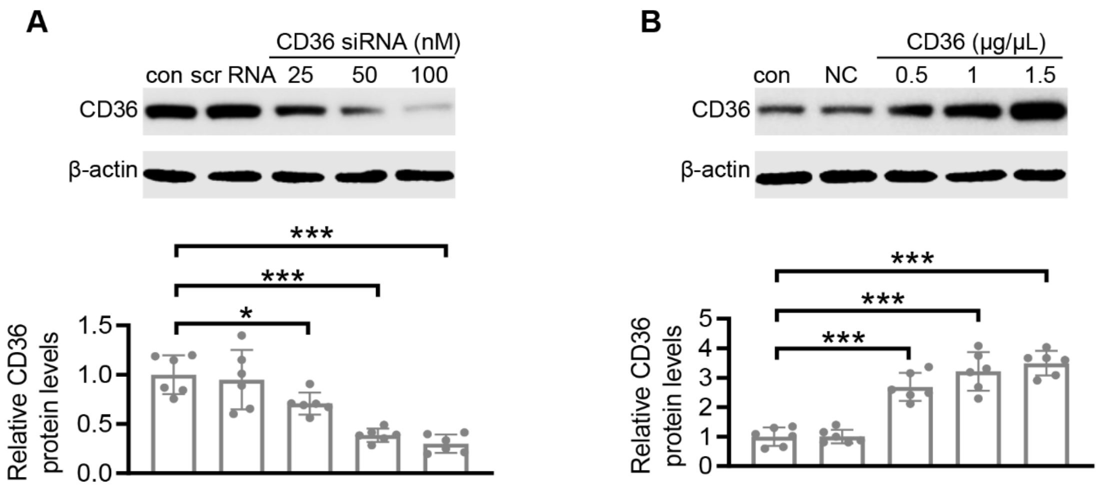

Supplementary Figure S4. Verification of *CD36* knockdown and overexpression in BMDMs (A) Western blot analysis was used to detect the efficiency of CD36 siRNA interference. (B) Transfection efficiency was verified after transfection with the CD36 overexpression plasmid. Therefore, a concentration of 50 nM siRNA and a concentration of 0.5 μg/μL plasmid were selected for further experiments (n=6, *P<0.05, ***P<0.001).

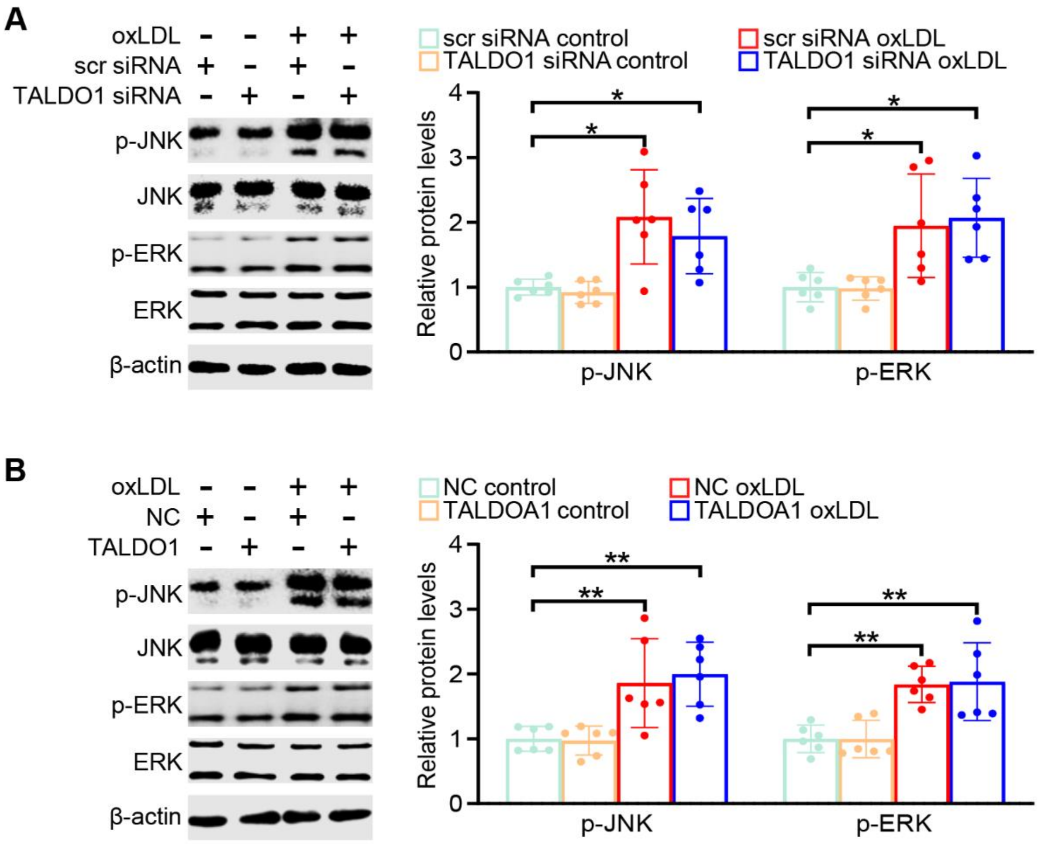

Supplementary Figure S5. Effects of transaldolase on the JNK and ERK signaling pathways (A) The expression levels of p-JNK and p-ERK in scr siRNA- and TALDO1 siRNA-transfected BMDMs after oxLDL (80 μg/mL) treatment for 48 h. (B) The expression levels of p-JNK and p-ERK in BMDMs from the indicated groups (*n*=6, *P<0.05, **P<0.01).

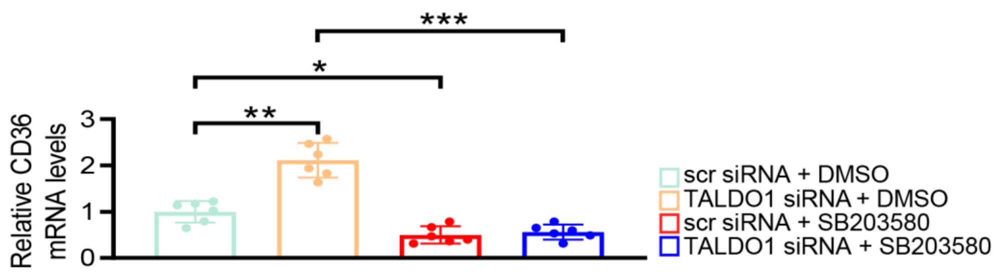

Supplementary Figure S6. The p-38 MAPK pathway is involved in transaldolase-mediated regulation of CD36 expression BMDMs were pre-incubated with the p38 inhibitor SB203580 (10 µM) for 3 h, followed by transfection with scr siRNA and TALDO1 siRNA prior to incubation with oxLDL (80 µg/mL) for 48 h. The CD36 mRNA expression was then determined (*n*=6, **P*<0.05, ***P*<0.01, ****P*<0.001).

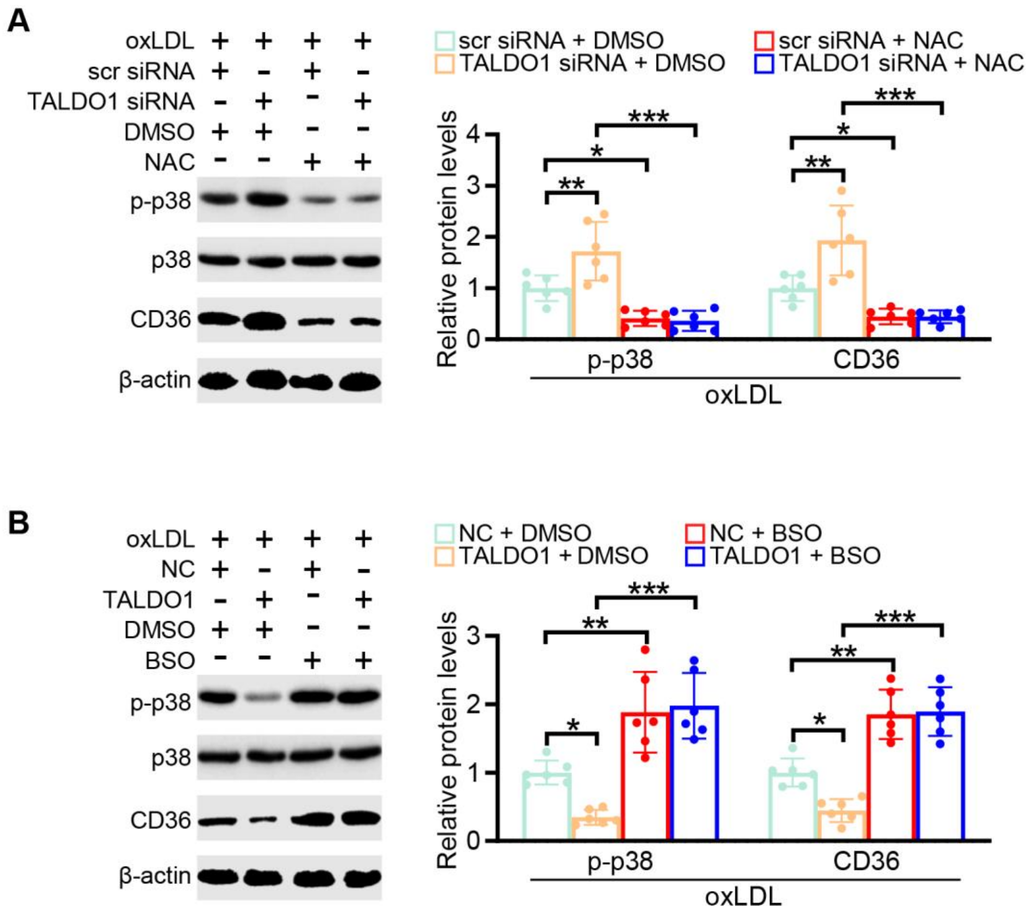

Supplementary Figure S7. GSH is involved in transaldolase-mediated foam cell formation (A) Scr siRNA- and TALDO1 siRNA-transfected BMDMs were pre-treated with or without N-acetyl cysteine (10 mM) for 16 h. The expression levels of p-p38 and CD36 in these cells were determined after oxLDL treatment for 48 h. (B) Representative western blot analysis of BMDMs pre-treated with or without buthionine sulfoximine (5 μM) for 16 h, followed by incubation with NC vector or TALDO1 plasmid prior to oxLDL (80 μg/mL) treatment for 48 h (n=6, *P<0.05, **P<0.01, ***P<0.001).
